# Characterization of the Arterial Anatomy of the Murine Hindlimb: Functional Role in the Design and Understanding of Ischemia Models

**DOI:** 10.1371/journal.pone.0084047

**Published:** 2013-12-30

**Authors:** Takashi Kochi, Yoshimichi Imai, Atsushi Takeda, Yukiko Watanabe, Shiro Mori, Masahiro Tachi, Tetsuya Kodama

**Affiliations:** 1 Department of Plastic and Reconstructive Surgery, Graduate School of Medicine, Tohoku University, Sendai, Miyagi, Japan; 2 Department of Plastic and Reconstructive Surgery, Tohoku University Hospital, Sendai, Miyagi, Japan; 3 Graduate School of Biomedical Engineering, Tohoku University, Sendai, Miyagi, Japan; 4 Department of Oral and Maxillofacial Surgery, Tohoku University Hospital, Sendai, Miyagi, Japan; Goethe University, Germany

## Abstract

**Rationale:**

Appropriate ischemia models are required for successful studies of therapeutic angiogenesis. While collateral routes are known to be present within the innate vasculature, there are no reports describing the detailed vascular anatomy of the murine hindlimb. In addition, differences in the descriptions of anatomical names and locations in the literature impede understanding of the circulation and the design of hindlimb ischemia models. To understand better the collateral circulation in the whole hindlimb, clarification of all the feeding arteries of the hindlimb is required.

**Objective:**

The aim of this study is to reveal the detailed arterial anatomy and collateral routes in murine hindlimb to enable the appropriate design of therapeutic angiogenesis studies and to facilitate understanding of the circulation in ischemia models.

**Methods and Results:**

Arterial anatomy in the murine hindlimb was investigated by contrast-enhanced X-ray imaging and surgical dissection. The observed anatomy is shown in photographic images and in a schema. Previously unnoticed but relatively large arteries were observed in deep, cranial and lateral parts of the thigh. The data indicates that there are three collateral routes through the medial thigh, quadriceps femoris, and the biceps femoris muscles. Furthermore, anatomical variations were found at the origins of the three feeding arteries.

**Conclusions:**

The detailed arterial anatomy of murine hindlimb and collateral routes deduced from the anatomy are described. Limitations on designs of ischemia models in view of anatomical variations are proposed. These observations will contribute to the development of animal studies of therapeutic angiogenesis using murine hindlimb ischemia models.

## Introduction

Therapeutic angiogenesis offers promise as a novel treatment for peripheral arterial diseases[1]–[7]. Appropriate development of ischemia models is required for successful studies of therapeutic angiogenesis. The purpose of therapeutic angiogenesis is to save the peripheral tissue by augmenting peripheral perfusion through the collateral vessels[6]–[9], thus, the treatment site should focus on the *‘choke vessel zone’* of the collateral routes [10]. In hindlimb ischemia models, several collateral routes, more than the medial-superficial thigh, are present [11]–[13]. Each collateral route is not related to *de novo* vessel growth but is part of the innate vasculature [3], [12], [14]. These observations suggest that accurate knowledge of vascular anatomy is essential to predict or understand the function of the collateral circulation. Despite its importance, to the best of our knowledge, there are no published reports in the literature describing the detailed vascular anatomy of the murine hindlimb. The majority of papers using murine ischemia models have paid attention only to the medial-superficial aspect of the thigh and have not considered the circulation of the whole hindlimb[4], [9], [11], [13], [15]–[26]. Since the thigh has great muscles surrounding the femur, the arteries previously described are not sufficient to feed the entire hindlimb. Considering the blood supply to all muscles in the thigh, it is logical to assume that undiscovered arteries must be present, probably medium sized arteries in the cranial, lateral and deep portions. These arteries must play an important role in collateral routes of the innate vasculature. In addition, though there are some reports giving explanations on the anatomy through the use of images and illustrations, the anatomical names and locations differ between reports[11], [12], [20]–[22], [26]–[28]. This lack of clarity impedes our understanding of the circulation in the hindlimb and makes the design of ischemia models uncertain. To gain a better understanding of the collateral circulation in whole hindlimb, the definitive identification of all the feeding arteries other than the femoral is required.

The aim of the present study was to map the detailed arterial anatomy and collateral routes in the murine whole hindlimb. The findings will permit the appropriate design of therapeutic angiogenesis studies and facilitate understanding of the circulation in ischemia models. The observed arterial anatomy is presented in high-resolution photographic images, not only of the medial-superficial region but also the deep and lateral aspects.

## Methods

All in vivo studies involving mice were carried out in strict accordance with the recommendations in the Guide for Proper Conduct of Animal Experiments and Related Activities in Academic Research and Technology, 2006. The protocol was approved by the Institutional Animal Care and Use Committee of Tohoku University (Permit Number: 2010BeA-029, 2011BeA-007, 2012BeA-016).

### Mice

Male BALB/c mice (10 animals, 10–27 weeks old, 26–33 g body weight) were used in this study. The mice were housed in the Animal Research Institute of Tohoku University Graduate School of Medicine under specific pathogen-free conditions, and had free access to food and water until the beginning of the experiments. All surgery and measurements were performed during inhalation anesthesia with 1.5–2.0% of isoflurane in air; great efforts were taken to minimize animal suffering.

### Contrast-Enhanced Vascular Imaging

In vivo contrast-enhanced X-ray imaging was performed using a micro-computed tomography (CT) scanner, newly developed specifically to image small experimental laboratory animals (LaTheta LCT-200, Hitachi-Aloka Medicals, Tokyo, Japan). Barium contrast agent was prepared according to the protocol described in previous reports [13], [27]. 5.0 mL of barium sulfate solution (Barytgen Sol, Fushimi Pharmaceutical Co. Kagawa, Japan) was mixed with 0.5 mL of heparin (Novo-Heparin, 5000 units/5 mL, Mochida Pharmaceutical Co., Tokyo, Japan), 0.5 mL of red acrylics (Liquitex; cadmium red medium, soft type, bonnyColArt Co.) and 0.2 g of gelatin (Cook gelatin, Morinaga & Co.) dissolved in 4.0 mL of warmed saline. The mixture was agitated just before injection. When mice (n = 2) were under deep general anesthesia, 0.1 mL heparin was administered intravenously and 0.6 mg papaverine (Papaverine hydrochloride, 40 mg/mL, Nichi-Iko, Toyama, Japan) subcutaneously. After 10 min, 2.5 mL of contrast medium was injected into the left ventricle through the thoracotomy, following transection of the caudal vena cava to permit drainage of blood. The perfused mice were scanned at resolutions of 24 µm and 120 µm pixel for plain angiography, and 48 µm pixel and a 96 µm slice thickness for CT. Acquired slice data were rendered as 3D images using a 3D analysis suite (Mimics, Materialize, Leuven, Belgium).

### Dissection of Hindlimb

Mice (*n = 8*) were dissected to investigate the arterial anatomy of the hindlimb. Arteries were dilated and fixed with red-colored resin for easier visualization and subsequent dissection. Before perfusion, the hindlimb hair was removed. Under deep general anesthesia, 0.1 mL heparin and 0.6 mg papaverine were administrated followed by thoracotomy and transection of the caudal vena cava. Subsequently, 2.5–5.0 mL of resin consisting of 40% chloroprene (Showa-Denko Chloroprene 671A, Showa-Denko, Tokyo, Japan), 10% red acrylics and 50% saline was injected into the left ventricle. The success of the perfusion was confirmed by observing marked dilation of the femoral and saphenous arteries. The perfused animals were stored in a refrigerator overnight to solidify the resin. The following day, 11 hindlimbs (8 left, and 3 right) from 8 mice were dissected to investigate the arterial anatomy, with the aid of a stereomicroscope (M80, Leica, Wetzlar, Germany). To clarify the anatomy of the nutrient arteries of the hindlimb, viscera of the caudal abdomen and pelvis, fat tissue, and veins were resected if at all possible. The routes and distributions of the arteries were recorded, together with any variations from the norm. The observed anatomy was photographed using digital cameras (Camedia C-5060, Olympus, Tokyo, Japan. istDs, Pentax, Tokyo, Japan). The terminologies used were compliant with the veterinary anatomy glossary established by the Japanese Association of Veterinary Anatomists, or with human anatomy [29].

## Results

### Angiography and Computed Tomographic Angiography of the Hindlimb

**Figure 1:**
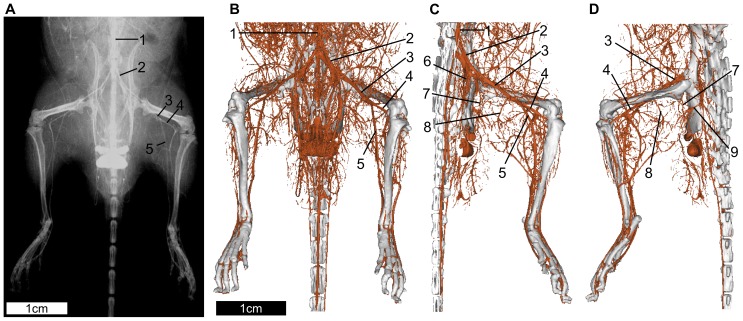
Angiography and CT angiography of the hindlimb. (**A**) Mouse caudal body angiogram. Though major vessels are delineated, their branches are not visualized. (**B**) 3D–CT of frontal view. While the threshold of the vascular image can be varied with CT-value (Hounsfield unit; HU), boxels with over 1000 HU are visualized as enhanced vessels. More vessels are visualized relative to angiography. (**C, D**) The ventral part of the abdomen and genitals are deleted to highlight better the vessels distributed to the hindlimb. C and D are medial and lateral views of the left hindlimb, respectively. Although a number of feeding branches can be detected, it is not sufficient to understand the detailed vascular anatomy. (1) aorta and vena cava, (2) external iliac artery and vein, (3) femoral artery and vein, (4) popliteal artery and vein, (5) saphenous artery and vein, (6) internal iliac artery and vein, (7) deep femoral artery and vein, (8) proximal caudal femoral artery and vein, (9) feeding vessels to the biceps femoris muscle.

First, we investigated the detailed vascular anatomy using high-resolution contrast-enhanced X-ray imaging (Figure 1). Major blood vessels, originating from the aorta, can be observed supplying the feet in plane angiography (Figure 1A). Though major and medium vessels were observed, small vessels could not easily be detected. Moreover, the 3D location could not be identified from the images. In order to investigate detailed vessel structures, 3D–CT was used (Figure 1B–1D). In 3D–CT, some branches of the iliac and femoral vessels were observed using depth information. However, their detailed distributions and the relationships between branches could not be identified accurately. Moreover, the arteries could not be distinguished from veins because both types of vessels were equally enhanced. Therefore, observation following fine surgical dissection was also required to provide an accurate picture of the detailed anatomy.

### Dissection of Hindlimbs

To investigate the detailed arterial anatomy, 11 hindlimbs from 8 mice were studied using surgical dissection. The procedures were performed on the pelvis and medial aspect of the thigh, and also on the deep layer and lateral aspect.

#### From the pelvis to the medial-superficial hindlimb

**Figure 2:**
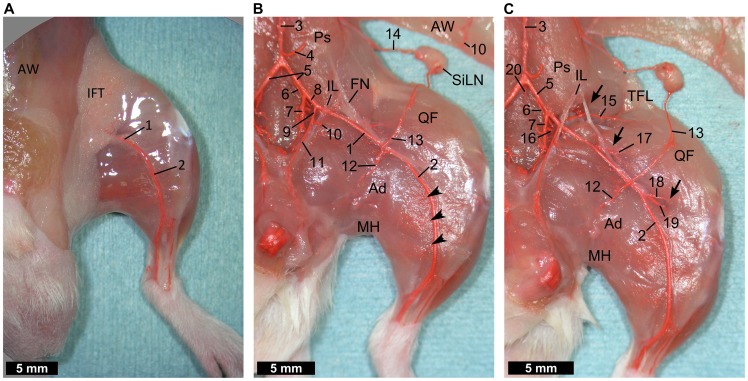
Dissection of the medial-superficial hindlimb. (**A**) After removal of the skin. A large part of the femoral artery is hidden by the inguinal fat tissue and overhanging abdomen. (**B**) After removal of the pelvic organs, fat tissue, and veins. The iliac artery and the entire femoral artery are exposed. Arteries distributed into the adductor muscles and medial hamstring muscles at mid (12) and distal portion (arrowheads) can also be seen. A minor branch to the medial thigh muscles can be observed but the nutrient vessels to muscles in the thigh and the popliteal artery cannot be seen in this view. (**C**) Parts of the iliopsoas muscle, quadriceps femoris muscle, and the adductor muscles are dissected (arrows) to expose the iliacofemoral artery (15), lateral circumflex femoral artery (17), and the popliteal artery (19). There are three feeding arteries to the quadriceps femoris muscle in this view; the iliacofemoral artery proximally, the medial proximal genicular artery distally, and the lateral circumflex femoral artery centrally. (1) femoral artery, (2) saphenous artery, (3) aorta, (4) caudal mesenteric artery, (5) common iliac artery, (6) cranial gluteal artery, (7) internal iliac artery, (8) external iliac artery, (9) pudendoepigastric trunk, (10) caudal epigastric artery, (11) external pudendal artery, (12) proximal caudal femoral artery, (13) superficial caudal epigastric artery, (14) illiolumbar artery, (15) iliacofemoral artery, (16) caudal gluteal artery, (17) lateral circumflex femoral artery, (18) medial proximal genicular artery, (19) popliteal artery, (20) median coccygeal artery, (Ad) adductor muscles, (AW) abdominal wall, (FN) femoral nerve, (IFT) inguinal fat tissue, (IL) inguinal ligament, (MH) medial hamstring muscles, (Ps) iliopsoas muscle, (QF) quadriceps femoris muscle, (SiLN) subiliac lymph node, (TFL) tensor fascia lata muscle.

Medial-superficial views of the left hindlimb are shown in Figure 2. After removal of the skin, a large part of the femoral artery was hidden by inguinal fat tissue and the overhanging abdomen (Figure 2A). To observe the intact arteries supplying the hindlimb together with its branches, lower abdominal and pelvic viscera were resected after laparotomy. Furthermore, fat tissue and veins were removed to facilitate visualization of the arteries. Thus, the iliac artery and the entire femoral artery were exposed (Figure 2B). The aorta splits into the left and right common iliac arteries and median coccygeal artery, just after giving off the caudal mesenteric artery. The common iliac artery runs caudal-laterally providing some branches, and then divides into the internal and external iliac arteries. The external iliac artery runs caudally and its nomenclature is changed to the femoral artery after it passes behind the inguinal ligament. The cranial gluteal artery arises from the common iliac artery while the caudal gluteal artery arises from the common or internal iliac artery, with individual variations. The cranial and caudal gluteal arteries run caudally and dorsally side-by-side and reach the dorsolumbar region. Just proximal to its crossing point with the inguinal ligament, the external iliac artery gives rise to the pudendoepigastric trunk, which is a short trunk that divides into the caudal epigastric artery and the external pudendal artery. The caudal epigastric artery is distributed to the abdominal wall, while the external pudendal artery runs medially, parallel to the inguinal ligament, and terminates in the perineal region. In the middle of the thigh, the proximal caudal femoral artery and the superficial caudal epigastric artery arise from the femoral artery as a very short common trunk or separately. The proximal caudal femoral artery runs medially on the surface of the adductor muscles, deep to the gracilis muscle, and is distributed to the superficial layer of the adductor muscles; the gracilis, pectineus, and adductor longus muscles, and the medial hamstring muscles; the semitendinosus and semimembranosus muscles. The superficial caudal epigastric artery courses laterally through the inguinal fat tissue and forms an anastomosis with the terminal branch of the illiolumbar artery around the subiliac lymph node. The saphenous artery descends on the medial surface of the leg, and divides into cranial and caudal branches. At the distal part of the medial thigh muscles, including the adductor and medial hamstring muscles, a significant distribution of small vessels arising from the saphenous artery was observed. While a minor branch to the medial thigh muscles was detected, large nutrient carrying vessels to the muscles in the thigh and the popliteal artery were not observed in this view. To expose the other nutrient vessels, parts of the iliopsoas, quadriceps femoris, and adductor muscles were dissected (Figure 2C). Behind the iliopsoas muscle, the iliacofemoral artery was shown to run transversely. The iliacofemoral artery arises from the distal end of the common iliac artery, dorsal to the internal iliac artery, and courses laterally sending branches to the surrounding muscles. Then the iliacofemoral artery descends into the proximal part of the quadriceps after sending branches to the gluteal muscles, including the tensor fascia lata. The femoral artery gives rise to the lateral circumflex femoral artery from its central portion. The lateral circumflex femoral artery has been called the anterior femoral artery. The lateral circumflex femoral artery dips in the central part of the quadriceps femoris muscle along with the muscular branch of the femoral nerve. The femoral artery bifurcates into the popliteal and saphenous arteries proximal to the knee and distal to the superficial caudal epigastric artery. The popliteal artery runs laterally between the quadriceps and adductor muscles toward the popliteal fossa. Near to the origin of the popliteal artery, the medial proximal genicular artery supplying the distal part of quadriceps femoris muscle was observed. Unequivocally, there are three supply routes to the quadriceps femoris muscle, the iliacofemoral artery proximally, the medial proximal genicular artery distally, and the lateral circumflex femoral artery centrally. Note however that in this viewing plane, the nutrient artery supplying the proximal and deep part of the medial thigh muscles could not be observed.

#### Deep layer of the medial thigh

**Figure 3:**
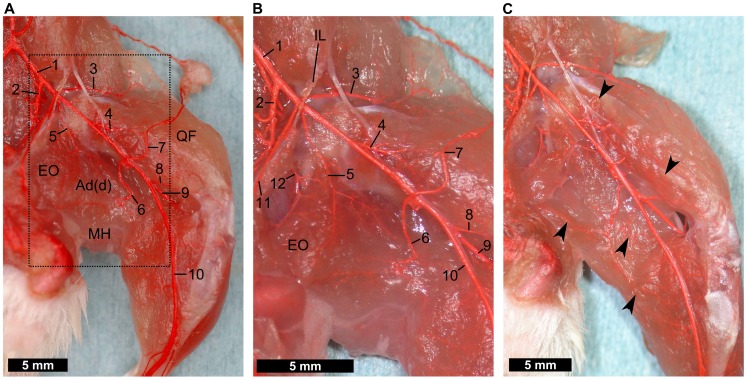
Dissection of the medial-deep hindlimb. (**A**) Superficial layer of the adductor muscles is removed. The deep femoral artery running distally and sending branches to the external obturator muscle, adductor muscles, and medial hamstring muscles can be seen (5). (**B**) Enlarged view of A. The external obturator muscle is divided from the pubis to expose the obturator artery (12). (**C**) Vascular networks in the quadriceps femoris muscle, medial hamstring muscles, and adductor muscles can be seen through the muscles (arrowheads). (1) common iliac artery, (2) internal iliac artery, (3) iliacofemoral artery, (4) lateral circumflex femoral artery, (5) deep femoral artery, (6) proximal caudal femoral artery, (7) superficial caudal epigastric artery, (8) medial proximal genicular artery, (9) popliteal artery, (10) saphenous artery, (11) external pudendal artery, (12) obturator artery, (Ad(d)) deep layer of adductor muscles, (EO) external obturator muscle, (MH) medial hamstring muscles, (QF) quadriceps femoris muscle.

The adductor muscles could easily be separated into the superficial and deep layers. The former includes the gracilis, pectineus, and adductor longus muscles, while the latter includes the adductor brevis and magnus muscles. Images after resection of the superficial layer are shown in Figure 3. Here, it can be clearly observed that the deep femoral artery runs on the deep layer of the adductor muscles, caudal to the femur (Figure 3A). The deep femoral artery ramifies into the proximal part of the adductor muscles, medial hamstrings, and the external obturator muscle (Figure 3B). End branches of the deep femoral artery reach the biceps femoris muscle through the medial hamstrings. Thus, medial thigh muscles also have three blood supply routes, the deep femoral artery proximally, proximal caudal femoral artery centrally, and small vessels from the saphenous artery and the popliteal artery distally. Clearly, microvascular networks are present in each muscle, connected to each nutrient vessel (Figure 3C).

#### Buttock and lateral hindlimb

**Figure 4:**
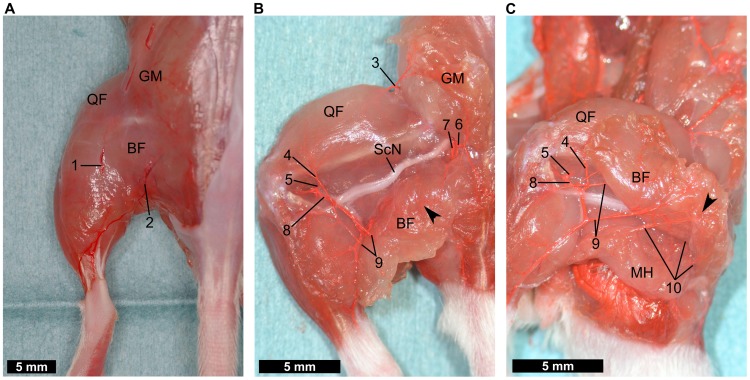
Dissection of the lateral hindlimb. (**A**) After removal of the skin. No large artery can be observed in this view. (**B**) Distal and cranial ends of the biceps femoris muscle are divided. Feeding arteries for the biceps femoris muscle from the cranial and caudal gluteal arteries are seen in the proximal region (6, 7). Near to the knee, the popliteal artery (5) sends branches to the distal portion of the quadriceps femoris muscle and the biceps femoris muscle (4, 9). (**C**) The distal and caudal margin of the biceps femoris muscle are divided. The terminal branches of the deep femoral artery reach the biceps femoris muscle through the medial hamstring muscles (10). The vascular network in the biceps femoris muscle can be seen in B and C (arrowheads). (1) lateral saphenous vein, (2) iscial vein, (3) iliacofemoral artery, (4) lateral proximal genicular artery, (5) popliteal artery, (6) cranial gluteal artery, (7) caudal gluteal artery, (8) distal caudal femoral artery, (9) branches to distal part of the biceps femoris muscle, (10) terminal branches of the deep femoral artery, (BF) biceps femoris muscle, (GM) gluteal muscles, (MH) medial hamstring muscles, (QF) quadriceps femoris muscle, (ScN) sciatic nerve.

Images of the gluteal region and the lateral hindlimb are shown in Figure 4. There was no notable artery on the surface of the lateral hindlimb, but two major veins were present, the lateral saphenous vein and the iscial vein, respectively (Figure 4A). The nutrient arteries supplying the biceps femoris muscle were observed behind the muscle (Figure 4B and 4C). In the gluteal region, the cranial and caudal gluteal arteries give rise to branches that supply the proximal part of the biceps femoris muscle; the cranial gluteal artery also supplies nutrient branches to the gluteal muscles. The caudal gluteal artery terminates in the internal pudendal artery and the lateral coccygeal artery. Near to the knee, the distal caudal femoral artery, from the popliteal artery, sends branches to the distal part of the biceps femoris. The distal caudal femoral artery also gives rise to the lateral proximal genicular artery which courses to the distal-lateral portion of the quadriceps femoris (Figure 4B). Several terminal branches of the deep femoral artery, dividing into the biceps femoris muscle through the medial hamstring muscles, were also detected (Figure 4C). Each nutrient vessel in the biceps femoris muscle seems to be connected to a microvascular network.

### Schema of Arterial Anatomy in Hindlimb

**Figure 5:**
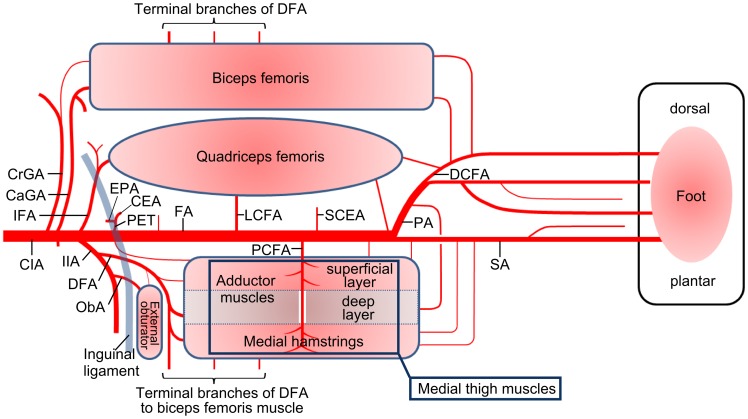
Schema of arterial anatomy in the hindlimb. Simplified developed illustration of the hindlimb arterial anatomy. When the femoral artery or iliac artery is obstructed, there are three, presumable collateral, routes through intramuscular networks in the quadriceps femoris muscle, biceps femoris muscle and the medial thigh muscles, including the medial hamstring muscles and adductor muscles. Moreover, it is noteworthy that the lateral circumflex femoral artery and the proximal caudal femoral artery locate at the central portion of the quadriceps femoris muscle and medial thigh muscles. (CaGA) caudal gluteal artery, (CEA) caudal epigastric artery, (CIA) common iliac artery, (CrGA) cranial gluteal artery, (DCFA) distal caudal femoral artery, (DFA) deep femoral artery, (EPA) external pudendal artery, (FA) femoral artery, (IFA) iliacofemoral artery, (IIA) internal iliac artery, (LCFA) lateral circumflex femoral artery, (ObA) obturator artery, (PA) popliteal artery, (PCFA) proximal caudal femoral artery, (PET) pudendoepigastric trunk, (SA) saphenous artery, (SCEA) superficial caudal epigastric artery.

Based on these observations, the arterial anatomy of the hindlimb is illustrated in a schema (Figure 5). The thigh is represented in development view divided at the caudal aspect, between the medial thigh and the biceps femoris muscles. There are three collateral routes through vascular networks in the quadriceps femoris, biceps femoris and medial thigh muscles. Each muscle, except the deep layer of the adductors, receives nutrient vessels mainly in three regions, proximally, distally, and centrally. Relatively large vessels from the iliac artery supply the proximal parts of each muscle, the cranial and caudal gluteal arteries to the biceps femoris, the iliacofemoral artery to the quadriceps femoris, and the deep femoral artery to the medial thigh muscles. The deep femoral artery also feeds the central part of the biceps femoris and the deep layer of the adductor muscles. The lateral circumflex femoral artery and the proximal caudal femoral artery, which arise individually from the femoral artery, are distributed into the central portions of the quadriceps femoris and the medial thigh muscles, except the deep layer of the adductor muscles. Vessels to the distal part of the medial thigh muscles from the saphenous artery are generally small, while arteries to the quadriceps and the biceps femoris from the popliteal or the distal caudal femoral arteries are relatively large.

### Anatomical Variations in Arteries

Anatomical variations were observed in various arteries significant for collateral circulation. Their routes and frequencies of occurrence were carefully recorded; the majority of variations occurred at the origin.

#### Variations in the origin of the caudal gluteal artery

**Figure 6:**
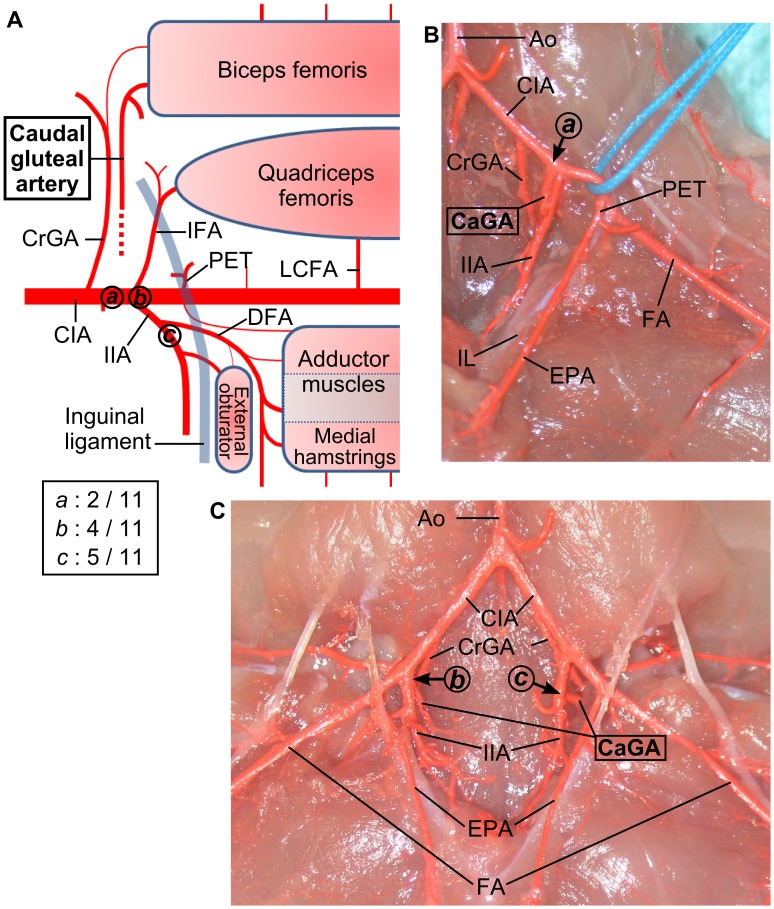
Anatomical variations in the origin of the caudal gluteal artery. (**A**) A schema of anatomical variations in the caudal gluteal artery. Three variations (variation ***a***, ***b***, and ***c***) in the origin of the caudal gluteal artery were observed. The originating points of the caudal gluteal artery are marked by circles ***a***, ***b***, and ***c***, respectively. Variation ***a*** have been observed in two limbs while ***b*** in four, and ***c*** in five. (**B**) An image of the variation ***a***. The caudal gluteal artery arose from the common iliac artery, just proximal to the internal iliac artery. (**C**) An image of the variation ***b*** on the right, and ***c*** left side. On the right side, the caudal gluteal artery arises from the medial-dorsal side of the origin of the internal iliac artery, while it arises from the internal iliac artery on the left side. (Ao) aorta, (CaGA) caudal gluteal artery, (CIA) common iliac artery, (CrGA) cranial gluteal artery, (DFA) deep femoral artery, (EPA) external pudendal artery, (FA) femoral artery, (IFA) iliacofemoral artery, (IIA) internal iliac artery, (IL) inguinal ligament, (LCFA) lateral circumflex femoral artery, (PET) pudendoepigastric trunk.

The caudal gluteal artery arose from the common iliac artery or the internal iliac artery (Figure 6). At its origin, three patterns of variations were observed. Two of them had origins in the common iliac artery, one was proximal to the bifurcation of the common iliac artery to the internal and external iliac arteries, and the other was dorsal to the bifurcation. The former was observed in 2 out of 11 limbs, while the latter in 4 limbs. Another variation had its origin in the internal iliac artery, observed in 5 limbs. These variations were observed not only between individuals, but also on both sides of the same mouse (Figure 6C).

#### Variations in the origin of the iliacofemoral artery

**Figure 7:**
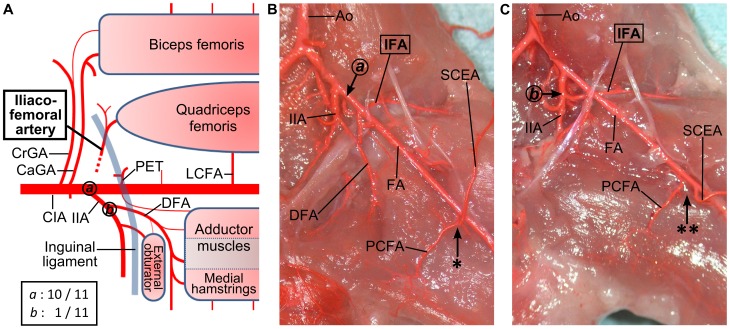
Anatomical variations in the origin of the iliacofemoral artery. (**A**) A schema of the anatomical variations in the iliacofemoral artery. In most limbs (10 out of 11), the iliacofemoral artery arose from the dorsal side of the origin of the internal iliac artery (circled ***a***). In only 1 limb, the iliacofemoral artery emerged as a branch of the internal iliac artery (circled ***b***). (**B**) An image of the variation ***a***. The iliacofemoral artery arises from the common iliac artery, dorsal to the internal iliac artery. (**C**) An image of variation ***b***. The internal iliac artery gives off the iliacofemoral artery instead of the common iliac artery. Additionally, the proximal caudal femoral artery and the superficial caudal epigastric artery arises from a short common trunk (* in B) in about half the limbs (5/11), while they arise separately (** in C) in the other half (6/11). (Ao) aorta, (CaGA) caudal gluteal artery, (CIA) common iliac artery, (CrGA) cranial gluteal artery, (DFA) deep femoral artery, (FA) femoral artery, (IFA) iliacofemoral artery, (IIA) internal iliac artery, (LCFA) lateral circumflex femoral artery, (PCFA) proximal caudal femoral artery, (PET) pudendoepigastric trunk, (SCEA) superficial caudal epigastric artery.

The iliacofemoral artery originated from the common iliac artery, dorsolateral to the origin of the internal iliac artery, in most cases (Figure 7A and 7B). But a variation originating from the internal iliac artery was observed in 1 limb (Figure 7C). In addition, variations in the origins of the proximal caudal femoral artery and the superficial caudal epigastric artery were also observed (Figure 7B and 7C). The proximal caudal femoral artery and superficial caudal epigastric artery formed a common trunk in about half the limbs but arose separately in the other half.

#### Variations in the origin of the deep femoral artery

**Figure 8:**
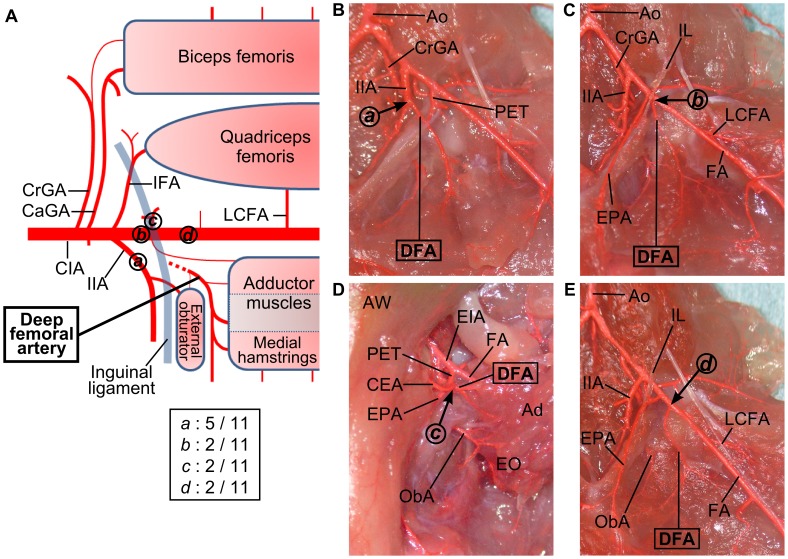
Anatomical variations in the origin of the deep femoral artery. (**A**) Schema of anatomical variations in the deep femoral artery. The deep femoral artery arises in a variable manner. In about half (5/11) of the limbs the deep femoral artery springs from the internal iliac artery (variation ***a***). The other variations had origins of the deep femoral artery from the external iliac artery (variation ***b***), pudendoepigastric trunk (variation ***c***), and the femoral artery (variation ***d***), in 2 out of 11 limbs each. (**B**) An image of the variation ***a***. The internal iliac artery is giving off the deep femoral artery. (**C**) An image of variation ***b***. The deep femoral artery arises from the medial-dorsal surface of the external iliac artery, just proximal to the pudendoepigastric trunk. (**D**) An image of the variation ***c***. The external obturator muscle and adductor muscles are lifted up. The deep femoral artery arises as a branch of the pudendoepigastric trunk. (**E**) An image of variation ***d***. The deep femoral artery arises from the femoral artery, distal to the pudendoepigastric trunk and inguinal ligament. Circled ***a***, ***b***, ***c***, and ***d*** indicate the originating points of the deep femoral artery in variations ***a***, ***b***, ***c*** and ***d***, respectively. (Ad) adductor muscles, (Ao) aorta, (AW) abdominal wall, (CaGA) caudal gluteal artery, (CEA) caudal epigastric artery, (CIA) common iliac artery, (CrGA) cranial gluteal artery, (DFA) deep femoral artery, (EIA) external iliac artery, (EO) external obturator muscle, (EPA) external pudendal artery, (FA) femoral artery, (IFA) iliacofemoral artery, (IIA) internal iliac artery, (IL) inguinal ligament, (LCFA) lateral circumflex femoral artery, (ObA) obturator artery, (PET) pudendoepigastric trunk.

The deep femoral artery arose in a variable manner (Figure 8). In about half (5/11) of the limbs the deep femoral artery originated from the internal iliac artery (Figure 8B). The other variations had their origins in the external iliac artery, pudendoepigastric trunk, and the femoral artery (Figure 8C–8E). These variations were observed in 2 out of 11 limbs.

## Discussion

In the present study, the arterial anatomy of the murine hindlimb has been studied in detail to provide a framework to understand its innate and collateral circulations. The observed anatomy has been clearly presented in high-resolution photographic images to avoid misunderstandings about the precise locations of each artery. In addition, anatomical variations in the arteries significant for collateral circulation have been noted. BALB/c mice were used in the present study while C57Bl/6 mice are commonly used in hindlimb ischemia studies, and BALB/c less frequently [14], [15], [18]. Previous studies have pointed out differences between strains in the degree of restoration of peripheral perfusion after ischemia [12], [15], [22]. Although possible differences in the degree of interarterial anastomosis have been suggested, obvious differences of arterial anatomy between strains have not been reported [12], [14], [20], [22].

Recently, detection of complex structures in small animals using sophisticated imaging techniques has been reported [30], [31]. Here, we have used a newly developed X-ray imaging system for small experimental animals to investigated the fine details of vascular anatomy. This system enabled us to observe the vascular structures in mice at high resolution, not only in plane images but also with depth information. As a result, though these methods were useful for the detection of already-known vessels, they were inadequate alone to investigate ‘*unknown*’ blood vessel anatomy. Therefore observations following surgical dissection were also required to reveal the exquisite fine details of arterial anatomy.

Because arteries in murine hindlimbs were too small to observe directly, treatments to visualize them were developed. We dilated and fixed arteries after perfusion with red-colored resin [32], [33]. Furthermore, veins and fat tissue were removed whenever possible to facilitate the viewing of fine arterial structures. When used in conjunction, these procedures made it relatively easy to determine the locations and trace the distributions of arteries. However, some vessels such as the iliacofemoral artery, deep femoral artery, and the popliteal artery, required additional muscular dissection for their routes to be readily determined. This is one of the reasons for missing them in the past and for the confusion about the arterial anatomy, as we occasionally come across, for example, descriptions of the location of the femoral bifurcation into the popliteal and saphenous arteries [11], [12], [20], [26], [27].

Our observations from detailed dissections have shown that the arterial anatomy in the murine hindlimb differs substantially from that of other animals, including the rabbit, but there are also similarities[8], [34]–[41]. For example, the iliac arteries, which arise from the aorta as the common iliac artery and divide into the internal and external iliac arteries, are similar to those in the human rather than other animals, in which the internal and the external iliac arteries arise from the aorta separately. Another example is the iliacofemoral artery and the lateral circumflex femoral artery, nutrient arteries supplying the proximal and central part of the quadriceps femoris muscle, which are also present in the horse [34], [36]. The lateral circumflex femoral artery, often termed the anterior femoral artery in the past, corresponds just to the descending branch of the lateral circumflex femoral artery in other animals [29]. The proximal caudal femoral artery is not seen in the horse or cattle or indeed humans but is present in dogs and cats. [37]–[40]. The proximal caudal femoral artery runs along the superficial layer of the adductor and medial hamstring muscles caudally, sending branches to the muscles and the skin. The proximal caudal femoral artery is often incorrectly termed “the deep femoral artery (*Arteria profunda femoris*)” [21], [22], [28]. However, this artery is distributed only into the superficial layer of the medial thigh and certainly not into the deep layer. This arrangement means that there has to be another artery supplying blood to the deep layer of the thigh. In fact, there is another artery, the deep femoral artery in this paper, which courses through the deep layer of the medial thigh, the biceps femoris, the hip joint and the femur; the same as the deep femoral artery in other animals. This artery has been noted in a few publications, but is called “the profunda” in only one, as far as we are aware [12], [20]. Nevertheless, according to its course and distribution, the artery that should be called the true deep femoral artery is not the former, but in fact the latter. The correct terminologies are vitally important because the deep femoral artery is the dominant nutrient artery supplying the thigh muscles, and is generally considered to be a key component of collateral routes. By calling the other artery “the deep femoral artery”, the presence of the true deep femoral artery will be at risk of not being considered. Missing the true deep femoral artery may lead to failure of studies using ischemia models. Over all, it has been demonstrated that there are three collateral routes through the medial thigh muscles, the quadriceps femoris muscle, and the biceps femoris muscle, when the femoral artery is obstructed surgically. These results are illustrated schematically in Figure 5.

It is noteworthy that collaterals have “side ways” on their central portion, the lateral circumflex femoral artery, proximal caudal femoral artery, and end branches of the deep femoral artery, respectively. In particular, the lateral circumflex femoral artery and the proximal caudal femoral artery are important, and must be considered in the design of ischemia models because they arise from the femoral artery as distinct branches, and can easily be manipulated during surgery. Surgical obstruction of the femoral artery, whether proximal or distal to the lateral circumflex femoral artery, will result in reverse directions of flow in the lateral circumflex femoral artery. And if the lateral circumflex femoral artery itself is obstructed, the collateral circulation will be ‘*confusingly different*’ without the knowledge garnered in the present investigation. The same reasoning applies to the proximal caudal femoral artery. Therefore, the lateral circumflex femoral artery and proximal caudal femoral artery can be used as markers in the design of ischemia models. At the same time, the collateral routes and their *‘choke vessel zone’* [10] in each model can be understood from the schema described in the present study.

In addition, anatomical variations in the arteries responsible for collaterals were also detected and mapped in the present inquiry. Variations were observed in the origins of the caudal gluteal artery, the iliacofemoral artery, and the deep femoral artery. The caudal gluteal artery and iliacofemoral artery arose from the common or internal iliac arteries, while the deep femoral artery originated from the internal iliac artery, external iliac artery, pudendoepigastric trunk, or the femoral artery. Variations in the deep femoral artery are significant when designing models of ischemia because they run from the internal iliac artery to the femoral artery, while the deep femoral artery is widely distributed. These variations may result in unexpected circulatory states even if the same surgery is performed. There may be other variations, especially in other murine strains. However, the arteries from the distal part of the common iliac artery to the proximal region of the femoral artery should be preserved to negate the effects of potential anatomical variations.

In conclusion, the detailed arterial anatomy of the murine hindlimb has been described. Various, previously unidentified, collateral arterial routes were deduced from the detailed arterial anatomy. Potential limitations on the design and interpretation of ischemia models produced by anatomical variations are highlighted. Future experiments will investigate the present differences between ischemia models developed by various procedures and their implications at the angiogenic and circulatory level. The mapping of the arterial network will provide a better understanding of the significance of changes in blood flow in the ischemic limb, both spontaneous and experimentally induced, and contribute to the development of models to investigate therapeutic angiogenesis in murine hindlimb ischemia models.
